# Impact of Preceding Flu-Like Illness on the Serotype Distribution of Pneumococcal Pneumonia

**DOI:** 10.1371/journal.pone.0093477

**Published:** 2014-04-01

**Authors:** Joon Young Song, Moon H. Nahm, Hee Jin Cheong, Woo Joo Kim

**Affiliations:** 1 Department of Pathology, School of Medicine, University of Alabama at Birmingham, Birmingham, Alabama, United States of America; 2 Department of Microbiology, School of Medicine, University of Alabama at Birmingham, Birmingham, Alabama, United States of America; 3 Division of Infectious Diseases, Department of Internal Medicine, Korea University College of medicine, Seoul, Republic of Korea; Institut Pasteur, France

## Abstract

**Background:**

Even though the pathogenicity and invasiveness of pneumococcus largely depend on capsular types, the impact of serotypes on post-viral pneumococcal pneumonia is unknown.

**Methods and Findings:**

This study was performed to evaluate the impact of capsular serotypes on the development of pneumococcal pneumonia after preceding respiratory viral infections. Patients with a diagnosis of pneumococcal pneumonia were identified. Pneumonia patients were divided into two groups (post-viral pneumococcal pneumonia versus primary pneumococcal pneumonia), and then their pneumococcal serotypes were compared. Nine hundred and nineteen patients with pneumococcal pneumonia were identified during the study period, including 327 (35.6%) cases with post-viral pneumococcal pneumonia and 592 (64.4%) cases with primary pneumococcal pneumonia. Overall, serotypes 3 and 19A were the most prevalent, followed by serotypes 19F, 6A, and 11A/11E. Although relatively uncommon (33 cases, 3.6%), infrequently colonizing invasive serotypes (4, 5, 7F/7A, 8, 9V/9A, 12F, and 18C) were significantly associated with preceding respiratory viral infections (69.7%, *P*<0.01). Multivariate analysis revealed several statistically significant risk factors for post-viral pneumococcal pneumonia: immunodeficiency (OR 1.66; 95% CI, 1.10–2.53), chronic lung diseases (OR 1.43; 95% CI, 1.09–1.93) and ICI serotypes (OR 4.66; 95% CI, 2.07–10.47).

**Conclusions:**

Infrequently colonizing invasive serotypes would be more likely to cause pneumococcal pneumonia after preceding respiratory viral illness, particularly in patients with immunodeficiency or chronic lung diseases.

## Introduction

Pneumonia is the leading cause of death in both children and adults worldwide, being estimated to affect approximately 450 million people in the world annually and to result in about 4 million deaths per year (7% of the world's yearly death) [1], [2]. A preceding viral infection significantly increases both the chance for bacterial pneumonia and the chance for death from pneumonia [3]. Many observational studies have found *Streptococcus pneumoniae* (pneumococcus) to be the predominant bacterial etiology of community-acquired pneumonia (CAP) in both adults and children [4].

Although it is a well-known pathogen, pneumococcus is an opportunistic pathogen and normally resides in the nasopharynx (NP) of a large fraction of a population. To survive in the NP as well as to be capable of causing invasive diseases, pneumococci express a variety of virulence factors that influence pneumococcal interactions with host cells and other bacterial species. These virulence factors include pneumolysin, pneumococcal surface protein A, pneumococcal surface protein C, pneumococcal surface adhesin A, and capsular polysaccharide. Capsular polysaccharide, which shields pneumococci from the host immune system, may be the most important of these virulence factors. It can increase virulence by more than a million fold in experimental invasive infections [5], and can also assist pneumococcal colonization within the NP [6], [7]. Pneumococci are known to express more than 90 serologically and biochemically distinct capsule types (serotypes), and various epidemiologic studies have found serotypes to be correlated with the propensity for a high rate of nasopharyngeal carriage or for invasive diseases [8].

Like other types of bacterial pneumonia, pneumococcal pneumonia is especially common following viral infections [9], [10]. Zhou et al.[10] reported the significant association of invasive pneumococcal pneumonia with the activities of influenza and respiratory syncytial virus. Pneumococci were obtained from 23.5% of lung cultures in autopsy cases during the 1918–1919 influenza pandemic [11], and from 10% of fatal cases during the 2009 influenza A/H1N1 pandemic [12]. Since secondary bacterial infection significantly increases the mortality associated with viral infections, many studies have investigated the synergistic mechanisms between viral and bacterial infections using various (animal) model systems. One group of studies found that viral infections lead to over-expression of pneumococcal binding receptors, impaired alveolar macrophage phagocytosis and neutrophil dysfunction [9], [13], [14]. These findings suggest that the host becomes susceptible to pneumococcal invasion into deeper tissues and develops pneumonia by micro-aspiration of pneumococci that are already colonizing the NP. Other studies found that viral infections make the host susceptible to pneumococci from other individuals, and they increase pneumococcal transmission among susceptible individuals [15], [16]. We hypothesized that infrequently colonizing invasive serotypes may cause post-viral pneumococcal pneumonia with enhanced transmission by preceding respiratory viral infection, or that frequently colonizing weakly invasive serotypes may cause post-viral pneumococcal pneumonia with successful tissue invasion in the susceptible host after preceding respiratory viral infection.

## Materials and Methods

### Collection of clinical data and pneumococcal isolates

Medical records from January 1, 2007 through December 31, 2011 were examined to select patient records with a discharge diagnosis of CAP at Korea University Guro Hospital (KUGH), a 1000-bed teaching hospital in Seoul, Korea. The clinical, radiological, and microbiological findings of all the selected records were re-evaluated to determine whether the patients fulfilled the following clinical and radiological criteria of CAP: (a) an acute pulmonary infiltrate evident on chest radiographs and consistent with pneumonia within 48 h after admission; (b) confirmatory findings on clinical examination; and (c) acquisition of the infection outside a hospital [17]. Patients with healthcare-associated pneumonia or hospital-acquired pneumonia were excluded [18].

The patients with CAP were determined to have pneumococcal pneumonia if their blood samples or adequate lower respiratory specimens yielded bacterial isolates that were optochin sensitive and had alpha hemolytic colonies in the clinical laboratory [19], [20], [21]. Adequate lower respiratory specimens included trans-bronchial aspirates, broncho-alveolar lavage (BAL) specimens, and sputum specimens with the predominant presence of gram-positive diplococci on a Gram stain of high-quality (>25 WBCs and <10 squamous epithelial cells/low-power field). All such bacterial isolates were presumptively identified as “pneumococci” and routinely stored at -80°C.

Two infectious disease doctors reviewed the medical records, and selected cases meeting the criteria of community-acquired pneumococcal pneumonia. Clinical data from the patients were obtained using a structured case report form, which included demographics, underlying diseases, time (month) of pneumonia development, the presence of bloodstream infection, the presence of a flu-like illness (FLI) in the recent 7 days before pneumonia development, results of multiplex respiratory viral PCR/culture, and 30-day case fatalities. This study was approved by the ethics committee of KUGH (IRB No. 2013-01-0037) and was conducted in accordance with the Declaration of Helsinki and Good Clinical Practice. The institutional review board waived written informed consent. Patient records/information was anonymized and de-identified prior to analysis.

### Definitions

Patients were defined as having post-viral pneumococcal CAP if they had FLI documented in their medical records 0–7 days before pneumonia development and were given respiratory viral tests (PCR or culture) at the time of admission. In KUGH, medical doctors have been regularly educated to perform respiratory viral tests, and document about respiratory symptoms with structured case report form if pneumonic patients have recent FLI. FLI was defined as sudden onset of fever (≥38°C) accompanied by ≥1 respiratory symptoms: cough, sore throat, or nasal symptoms. All the other cases were defined as having primary pneumococcal CAP. Influenza epidemic periods were defined based on Korean Influenza & Respiratory Virus Scheme (KINRESS), which reports an influenza index (influenza-like illness cases among 1000 patients) weekly, and declares influenza epidemic if the influenza-like illness index is higher than the upper limit of the mean influenza-like illness index ±2 standard deviations of the non-epidemic periods of the previous 3 years [22], [23]. Influenza epidemic period is defined to end if the influenza index decline below the upper limit above mentioned for four consecutive weeks.

### Serotyping of pneumococcal isolates

All the “pneumococcal” isolates were recovered from the clinical laboratory and were re-identified in the research laboratory by colony morphology, optochin susceptibility, and bile solubility testing. Following the re-identification, all the isolates with appropriate properties were serotyped with the multibead serotyping assay method described previously [24], which included multibead assay with monoclonal antibodies (reaction A) and multibead assay with multiplex PCR (reaction B and C). The assay has been designed to identify the pneumococcal capsular PSs of all 93 serotypes and non-typeable (NT) pneumococci. NT pneumococci were classified as Group I or Group II, and Group II pneumococci were further divided into null capsule clade (NCC) 1, 2 and 3 [25].

Based on the literature review of carriage rates, carriage duration, and invasive disease potential[8], [26], [27], [28], [29], [30], each serotype was classified into four groups: infrequently colonizing but invasive (ICI) serotypes (1, 4, 5, 7F, 8, 9V/9A, 12F, and 18C), frequently colonizing and invasive (FCI) serotypes (3, 14, and 19A), frequently colonizing but weakly invasive (FCWI) serotypes (6A, 6B, 11A/11E, 15F/15A, 15B, 15C, 16F, 19F, 23F, and 35B), and serotypes that have not yet been classified (6C, 6D, 7B/7C/40, 9N, 10A/39, 10B, 13, 17F/17A, 20, 22F/22A, 23A, 24F/24A/24B, 25F/25A/38, 28F/28A, 31, 33F/33A/37, 34, 35F/47F, 35A/35C/42, 36, 41F/41A, 45, and NT).

### Statistical analysis

We performed case-control analysis to compare the demographics, clinical characteristics, and serotype distributions of post-viral pneumococcal pneumonia and primary pneumococcal pneumonia. We also investigated the association between serotype distribution and influenza epidemics. Data were analyzed using SPSS version 12.0 (SPSS Inc., Chicago, IL, USA). For categorical data, univariate analysis was performed using either the chi-square test or Fisher's exact test. The Mann-Whitney *U* test was used to compare ages between two groups and was expressed as a median (interquartile range, IQR). *P*<0.05 was considered to be statistically significant. Multivariate analysis was carried out to assess independent risk factors of post-viral pneumococcal pneumonia using a logistic regression model.

## Results

### Demographic and clinical characteristics: post-viral pneumococcal pneumonia versus primary pneumococcal pneumonia

Although the review of medical records identified 930 pneumococcal CAP cases, 11 cases were excluded because the research laboratory found that the associated bacterial isolates were non-pneumococcal streptococci. The remaining bacterial isolates from 919 patients were positive for *lytA* on polymerase chain reaction (reaction B). Urinary antigen test (BinaxNOW *S. pneumoniae* assay) was taken in 835 cases, and 697 among 835 cases (83.5%) showed positive results. The included 919 cases had a median age of 63 (interquartile range, 44–72) years and were comprised of more male (66.4%) than female patients (Table 1). Bacteremic pneumonia was found in 19 (2.1%) patients, and 100 (10.9%) patients died within 30 days of developing pneumonia.

**Table 1:** Comparison of patient characteristics and clinical outcomes of post-viral pneumococcal pneumonia and primary pneumococcal pneumonia.

	Total (*n* = 919)	Post-viral pneumococcal pneumonia (*n* = 327)	Primary pneumococcal pneumonia (*n* = 592)	*P* value
Age, median (IQR)	63 (44–72)	66 (52–75)	61 (42–71)	<0.01
Age group, No. (%)				<0.01
≤5 years	102 (11.1)	31 (9.5)	71 (12.0)	
6–18 years	22 (2.4)	3 (0.9)	19 (3.2)	
19–49 years	144 (15.7)	42 (12.8)	102 (17.2)	
50–64 years	239 (26.0)	76 (23.2)	163 (27.5)	
≥65 years	412 (44.8)	175 (53.5)	237 (40.0)	
Gender, male No.(%)	610 (66.4)	228 (69.7)	382 (64.5)	0.13
Underlying diseases, No. (%)	604 (65.7)	244 (74.6)	360 (60.8)	<0.01
Immunodeficiency*	113 (12.3)	56 (17.1)	57 (9.6)	<0.01
Chronic lung disease†	376 (40.9)	160 (48.9)	216 (36.5)	<0.01
Chronic cardiovascular disease‡	296 (32.2)	112 (34.3)	184 (31.1)	0.34
Chronic liver disease§	101 (11.0)	45 (13.8)	56 (9.5)	0.05
Chronic renal disease¶	46 (5.0)	17 (5.2)	29 (4.9)	0.88
Diabetes mellitus	164 (17.8)	64 (19.6)	100 (16.9)	0.32
Cases during influenza epidemic periods, No. (%)	493 (53.6)	177 (54.1)	316 (53.4)	0.84
Laboratory-confirmed influenza, No. (%)	23 (2.5)	23 (7.0)	0 (0)	-
Laboratory-confirmed viral infection, No. (%)	44 (4.8)	44 (13.5)	0 (0)	-
Bacteremia, No. (%)	19 (2.1)	9 (2.8)	10 (1.7)	0.33
30-day mortality, No. (%)	100 (10.9)	40 (12.2)	60 (10.1)	0.38

*Patients who are immunocompromised due to either medications or immune disorders including human immunodeficiency syndrome.

† Patients who have asthma, chronic obstructive pulmonary disease, or bronchiectasis.

‡ Patients who have heart failure, cardiomyopathy, or anther chronic condition affecting cardiac function.

§ Patients who have either chronic hepatitis or cirrhosis; chronic hepatitis was defined as an elevation of serum transaminases above 1.5 times of upper normal limit for longer than 6 months, and cirrhosis was defined by the presence of biochemical and radiological findings consistent with cirrhosis.

¶ Patients who have either nephrotic syndrome or chronic renal failure.

IQR, interquartile range.

The majority of cases (592 patients, 64.4%) had primary pneumococcal pneumonia (Table 1), but a small but significant number of cases (327 patients, 35.6%) had post-viral pneumococcal pneumonia. In patients with post-viral pneumococcal pneumonia, cough, rhinorrhea/nasal stuffiness and sore throat were observed in 64.5% (211 patients), 26.9% (88 patients) and 58.1% (190 patients), respectively. Patients with post-viral pneumococcal pneumonia were older than those with primary pneumococcal pneumonia (*P*<0.01); more than half (53.5%) of the patients with post-viral pneumococcal pneumonia were 65 years old or older. Underlying medical diseases were more common in patients with post-viral pneumococcal pneumonia compared to those with primary pneumococcal pneumonia (74.6% versus 60.8%, *P*<0.01); immunodeficiency (*P*<0.01), chronic lung diseases (*P*<0.01), and chronic liver diseases (*P* = 0.05) were observed most often among patients with post-viral pneumococcal pneumonia. Four hundred and ninety-three of the cases (53.6%) occurred during influenza epidemic periods without a significant difference between post-viral and primary pneumococcal pneumonia (*P* = 0.84). The two groups had similar rates of bacteremia and 30-day mortality.

Respiratory viruses were actually isolated from 44 (13.5%) of the post-viral pneumococcal pneumonia patients (Table 2).

**Table 2:** Serotype distribution of pneumococcal pneumonia based on laboratory-confirmed viral infections.

Virus types (No.)	Serotypes (No.)
Influenza virus (23)	3 (4), 4 (1), 5 (1), 6A (2), 6C (1), 6D (1), 7F/7A (1), 8 (1), 9V/9A (1), 11A/11E (1), 12F (1), 14 (1), 19F (1), 19A (1), 34 (1), 35B (2), NT (2)
Respiratory syncytial virus (7)	9V/9A (2), 19F (1), 19A (2), 23F (1), NT (1)
Parainfluenza virus (7)	3 (1), 4 (1), 6B (1), 14 (1), 19F (1), 19A (1), 34 (1)
Metapneumo virus (2)	6D (1), 17F/17A (1)
Adenovirus (3)	19A (1), 23F (1), NT (1)
Rhinovirus (2)	10A/39 (1), 13 (1)

### Comparison of serotype distributions: post-viral pneumococcal pneumonia versus primary pneumococcal pneumonia

**Figure 1:**
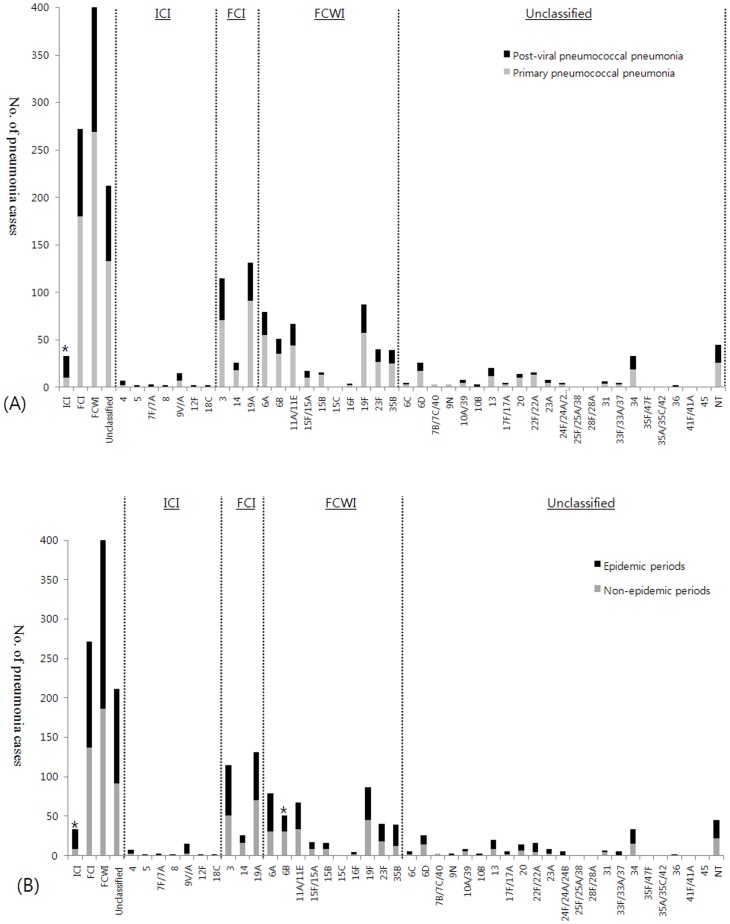
Serotype distribution of *Streptococcus pneumoniae* was analyzed regarding preceding respiratory viral infections and influenza epidemic periods. (A) Serotype distribution of *Streptococcus pneumoniae* in terms of preceding respiratory viral infections among patients with pneumonia. ICI serotypes (4, 5, 7F/7A, 8, 9V/9A, 12F and 18C) were more likely to cause post-viral pneumococcal pneumonia compared to other serotypes (*P<0.05). (B) Serotype distribution of *Streptococcus pneumoniae* in terms of influenza epidemic periods among patients with pneumonia. ICI serotypes were more likely to cause pneumococcal pneumonia during influenza epidemic periods, while serotype 6B was more common during non-epidemic periods (*P<0.05). ICI, infrequently colonizing but invasive serotypes; FCI, frequently colonizing and invasive serotypes; FCWI, frequently colonizing but weakly invasive serotypes; NT, non-typeable.

When pneumococcal serotypes are grouped according to categories, most pneumococcal CAP cases were associated with pneumococci of FCI (272 cases, 29.6%), FCWI (402 cases, 43.7%) and unclassified (212 cases, 23.1%) serotypes, but a small number of cases (33 cases) were due to ICI serotype pneumococci (Table 3). When the two patient groups were compared (Table 2 and Fig. 1A), the distribution of FCI, FCWI, and unclassified serotypes did not differ between them, but ICI serotypes were observed more often among patients with preceding respiratory viral infections than among those with primary pneumococcal pneumonia (23 cases versus 10 cases, *P*<0.01). In addition, we compared 44 PCR-confirmed viral cases versus 592 FLI-negative primary pneumococcal pneumonia cases. Likewise, ICI serotypes were significantly associated with preceding respiratory viral infections (*P*<0.01): ICI serotypes (60.0%, 15 among 25 cases), FCI serotypes (6.3%, 12 among 192 cases), FCWI serotypes (3.9%, 11 among 280 cases) and unclassified serotypes (4.3%, 6 among 139 cases). In the age-stratified analyses, ICI serotypes were rare among the pediatric patients, and statistically significant difference was not evaluable. When we restrict the cases to the elderly aged ≥50 years, the results were the same as those from all study subjects (data not shown).

**Table 3:** Comparison of serotype distribution of post-viral pneumococcal pneumonia and primary pneumococcal pneumonia.

Serotype, No. (%)		Post-viral pneumococcal pneumonia	Primary pneumococcal pneumonia	*P* value
ICI serotypes		23 (69.7)	10 (30.3)	<0.01
FCI serotypes		92 (33.8)	180 (66.2)	0.50
FCWI serotypes		133 (33.1)	269 (66.9)	0.17
Unclassified serotypes		79 (37.3)	133 (62.7)	0.57
ICI serotypes	4	5 (71.4)	2 (28.6)	0.10
	5	2 (100)	0 (0)	0.13
	7F/7A	3 (100)	0 (0)	0.05
	8	2 (100)	0 (0)	0.13
	9V/9A	8 (53.3)	7 (46.7)	0.42
	12F	2 (100)	0 (0)	0.13
	18C	1 (50.0)	1 (50.0)	0.54
FCI serotypes	3	44 (38.3)	71 (61.7)	0.53
	14	8 (30.8)	18 (69.2)	0.68
	19A	40 (30.5)	91 (69.5)	0.20
FCWI serotypes	6A	24 (30.4)	55 (69.6)	0.33
	6B	16 (31.4)	35 (68.6)	0.55
	11A/11E	23 (34.3)	44 (65.7)	0.90
	15F/15A	7 (41.2)	10 (58.8)	0.62
	15B	3 (18.8)	13 (81.3)	0.19
	15C	0 (0)	1 (100)	0.46
	16F	2 (50.0)	2 (50.0)	0.13
	19F	30 (34.5)	57 (65.5)	0.91
	23F	13 (32.5)	27 (67.5)	0.74
	35B	14 (35.9)	25 (64.1)	0.73
Unclassified serotypes	6C	2 (40.0)	3 (60.0)	0.84
	6D	9 (34.6)	17 (65.4)	0.92
	7B/7C/40	0 (0)	3 (100)	0.56
	9N	0 (0)	3 (100)	0.56
	10A/39	3 (37.5)	5 (62.5)	0.91
	10B	2 (66.7)	1 (33.3)	0.29
	13	8 (40.0)	12 (60.0)	0.65
	17F/17A	2 (40.0)	3 (60.0)	0.84
	20	4 (28.6)	10 (71.4)	0.78
	22F/22A	3 (18.8)	13 (81.3)	0.19
	23A	3 (37.5)	5 (62.5)	0.91
	24F/24A/24B	2 (40.0)	3 (60.0)	0.84
	25F/25A/38	0 (0)	1 (100)	0.46
	28F/28A	0 (0)	1 (100)	0.46
	31	2 (33.3)	4 (66.7)	0.64
	33F/33A/37	2 (40.0)	3 (60.0)	0.84
	34	14 (42.4)	19 (57.6)	0.46
	35F/47F	1 (100)	0 (0)	0.36
	35A/35C/42	1 (100)	0 (0)	0.36
	36	2 (100)	0 (0)	0.13
	41F/41A	1 (100)	0 (0)	0.36
	45	0 (0)	1 (100)	0.46
	Nontypeable	19 (42.2)	26 (57.8)	0.34
	Group I	19	21	-
	Group II	0	5*	-
Total		327 (35.6)	592 (64.4)	-

*Two null capsule clade (NCC) 1 isolates, one NCC2 isolate, and two NCC3 isolates.

ICI, infrequently colonizing but invasive serotypes; FCI, frequently colonizing and invasive serotypes; FCWI, frequently colonizing but weakly invasive serotypes.

When individual pneumococcal capsule types were compared between the two patient groups, 7F/7A was more common among post-viral pneumonia patients than among primary pneumococcal CAP patients (*P* = 0.05) (Table 3). Serotypes 3 and 19A were the most common serotypes found in both patient populations, and viral etiology could not be associated with specific serotypes of pneumococci (Table 2). Taken together, it was difficult to identify a serotype that is more common in one group than another. Forty-five cases (4.9%) were caused by NT pneumococci, but most of them (88.9%) belonged to Group I, which has defective *cps* (Table 3). The remaining five NT isolates belonged to Group II: two isolates of NCC1, one isolate of NCC2, and two isolates of NCC3. Contrary to NT carriage isolates, which usually lack the capacity to produce a capsule (Group II) [31], most of the NT pneumococci from pneumonia cases belonged to Group I in the present study.

To estimate the vaccine coverage rate, the pneumococcal serotypes were classified according to the serotypes included in the three different pneumococcal vaccines. The overall coverage rates of 7-valent pneumococcal conjugate vaccine (PCV7), PCV13 and 23-valent pneumococcal polysaccharide vaccine (PPV23) were 24.8%, 60.7% and 63.3% respectively (Table S1 in File S1). The PCV7 would provide protection against 24.5% (80) of the post-viral pneumococcal pneumonia cases compared with 25.0% (148) of the primary pneumococcal pneumonia cases (*P* = 0.87). Similarly, PCV13 would provide protection against 59.0% (193) of the post-viral pneumococcal pneumonia cases versus 61.7% (365) of the primary pneumococcal pneumonia cases (*P* = 0.44), and the PPV23 would provide protection against 62.4% (204) of the post-viral pneumococcal pneumonia cases versus 63.9% (378) of the primary pneumococcal pneumonia cases (*P* = 0.67). Thus, the two patient groups would be comparably protected by all the vaccines.

### Risk factors for post-viral pneumococcal pneumonia

The results of multivariate logistic regression analysis for risk factors associated with post-viral pneumococcal pneumonia are summarized in Table 4. Immunodeficiency (OR 1.66; 95% CI, 1.10–2.53), chronic lung diseases (OR 1.43; 95% CI, 1.09–1.93) and ICI serotypes (OR 4.66; 95% CI, 2.07–10.47) were considered as independent risk factors for post-viral pneumococcal pneumonia.

**Table 4:** Multivariate logistic regression analysis for risk factors of post-viral pneumococcal pneumonia.

Variables	Odds ratio (95% confidence interval)	*P* value
Age group	(Reference: ≤5 years)	
6–18 years	0.38 (0.10–1.37)	0.14
19–49 years	0.76 (0.43–1.35)	0.35
50–64 years	0.79 (0.47–1.35)	0.39
≥65 years	1.25 (0.76–2.07)	0.38
Immunodeficiency	1.66 (1.10–2.53)	0.02
Chronic lung diseases	1.43 (1.09–1.93)	0.02
Chronic liver diseases	1.30 (0.83–2.03)	0.26
Degree of colonization	(Reference: unclassified serotypes)	
ICI serotypes	4.66 (2.07–10.47)	<0.01
FCI serotypes	0.97 (0.66–1.41)	0.85
FCWI serotypes	0.95 (0.66–1.35)	0.76
Influenza epidemic periods	0.99 (0.74–1.31)	0.92

ICI, infrequently colonizing but invasive serotypes; FCI, frequently colonizing and invasive serotypes; FCWI, frequently colonizing but weakly invasive serotypes.

### Serotype distribution of pneumonia cases in relation to influenza epidemic periods: epidemic periods versus non-epidemic periods

Considering that influenza is the most common cause of respiratory viral infections that precede cases of pneumonia, we additionally compared the distribution of the pneumococcal serotypes of pneumonia cases between influenza epidemic periods and non-epidemic periods (Fig. 1B and Table S2 in File S1). Pneumococcal pneumonia of ICI serotypes occurred predominantly during influenza epidemic periods (75.8%, *P* = 0.01), whereas other serotypes (FCI, FCWI and unclassified) were evenly distributed between epidemic and non-epidemic periods.

## Discussion

Bacterial pneumonia often follows respiratory viral infections and can be lethal in some patients. The mechanisms involved in the synergism between viral infections and bacterial pneumonia have been extensively investigated in animal models and have produced evidence for increased invasiveness as well as transmission following respiratory viral infections. Increased transmissibility has been associated with prolonged shedding and wider spreading of pneumococci in a strain- or serotype-dependent manner after respiratory viral infections [16]. In the mouse model, influenza-neutralizing monoclonal antibodies inhibited pneumococcal transmission [15]. Increased invasiveness has been associated with the paradoxical suppression of the host immune system or with enhanced bacterial adherence. Alveolar macrophage function is impaired by dysregulated cytokine responses, while neutrophil function is suppressed as the amount of neutrophil-activating chemokines diminishes [9]. Meanwhile, pneumococcal adherence is facilitated by the up-regulation of pneumococcal binding receptors: fibronectin, glycoproteins of basal progenitor cells, polymeric immunoglobulin receptor (pIgR), and platelet-activating factor receptor (PAFR) [9]. In addition, pneumococcal binding to pIgR and PAFR may facilitate the development of bacteremic pneumonia [32], [33].

If increased invasiveness is dominant in human, we should have observed increases in pneumonia due to the relative non-invasive capsule types that commonly colonize the NP. Instead, we found that ICI serotypes (4, 5, 7F/7A, 8, 9V/9A, 12F, and 18C) occurred more frequently among cases of post-viral pneumococcal pneumonia. This observation strongly suggests that increased transmissibility may be the critical factor in humans. However, our findings do not imply that changes that increase invasion are irrelevant. Such changes may have assisted infections by ICI serotypes.

In addition to serotype distribution based on preceding respiratory viral infections, we compared serotype distribution between influenza epidemic periods and non-epidemic periods considering that influenza viruses circulate in Korea during confined periods, usually between November to April [22], [23]. As expected, ICI serotypes were more prevalent during influenza epidemic periods than during non-epidemic periods.

The serotypes associated with (epidemic) outbreaks of pneumococcal pneumonia have been reported to be serotypes 1, 4, 5, 7F, 8, 9, and 12F [34], [35], [36], [37]. An interesting observation is that these serotypes are almost identical to the serotypes associated with pneumonia cases following respiratory viral infections. Previous studies of outbreaks of pneumococcal pneumonia have not provided information on any respiratory viral infections that may have preceded the outbreaks. Thus, epidemic pneumococcal pneumonia outbreaks might be related to concurrent respiratory viral infections. Consequently, surveillance of respiratory viral infections in relation to pneumococcal outbreaks would be warranted and should be performed with highly sensitive methods (PCR or real-time PCR) considering the limited viral shedding periods [38].

To identify the population(s) at greatest risk of pneumococcal pneumonia after preceding respiratory viral infections, we investigated post-viral pneumococcal pneumonia patients for risk factors. Immunodeficiency and chronic lung diseases increased the risk for post-viral pneumococcal pneumonia by 66% and 43%, respectively. Increased pneumococcal pneumonia in immune-compromised patients may be the result of defective T regulatory cell and immunomodulatory responses [39]. In addition, it is well known that viral shedding persists longer in the immunocompromised patients than in healthy adults [40], [41]. Prolonged viral shedding and susceptible time periods might contribute to the development of concomitant or secondary pneumococcal pneumonia. As for chronic lung diseases, acute exacerbation of asthma and chronic obstructive pulmonary disease (COPD) are triggered by respiratory viral infections, which may increase the risk of secondary bacterial infection. Impaired interferon and Th1 responses result in uncontrolled viral replication and exaggerated inflammatory responses in asthmatic patients [42], and systemic corticosteroids for asthma or COPD exacerbations may be associated with slower viral clearance [43]. However, patients with immunodeficiency or chronic lung diseases are also susceptible to flu, so prospective cohort studies are required to clarify if these are real risk factors for pneumococcal pneumonia after respiratory viral infections.

30-day mortality did not differ between the two groups (post-viral pneumococcal pneumonia versus primary pneumococcal pneumonia). Given the older age and co-morbidities among the post-viral pneumonia cases, this group would be expected to show higher mortality. Interestingly, the ICI serotypes are associated with less mortality than serotypes 3 and 19A [44]. This might account for similar mortality in the two groups.

Pneumococcal vaccination is a part of the influenza pandemic preparedness plan. In our study, serotypes 3 and 19A were the most common, followed by serotypes 19F and 6A. Since serotypes 3, 19A, and 6A are included in PCV13, PCV13 could be much better than PCV7 and be useful as a part of the preparedness plan. However, the immunogenicity of PCV13 appears to be low with serotype 3 [45], [46], and PCV13 does not include serotypes 8 and 12F, which are represented in PPV23. Actually in the study by Sanz et al. [47], the introduction of pediatric conjugate vaccine (PCV7) led to overall decrease in invasive pneumococcal diseases, but those by non-vaccine serotypes (particularly serotype 8) increased markedly in HIV-infected patients. Thus, adults aged ≥19 years with immunocompromising conditions should receive a dose of PCV13 first, followed by a dose of PPV23 with ≥8 week interval [48]. In addition, due to its broader serotype coverage, PPV23 may be preferable to PCV13 for the elderly aged 65 years or more (Table S1 in File S1) [49].

This study has several limitations. First, this study had a retrospective design. To increase its accuracy, we combined clinical FLI criteria and respiratory viral tests in defining preceding respiratory viral infections. Considering that influenza patients with pneumonia are less likely to have nasal symptoms compared to those without pneumonia [50], we did not limit the post-viral pneumonia group to those who have nasal symptoms. Because respiratory viral tests were performed at the time of pneumonia diagnosis, the isolation rate of respiratory viruses (13.4%) was relatively low in the present study. Patients are at great risk for pneumococcal pneumonia at 5–7 days after influenza infection [51], [52], but influenza viral shedding is known to persist less than 7 days in general [38]. Moreover, influenza viruses may bind better to α2-3-linked receptors rather than α2-6-linked receptors in pneumonic patients, resulting in low viral isolation from upper respiratory specimens. In human, sialic acid (SA)-α2,6Gal is dominant on epithelial cells in nasal mucosa, while SA-α2,3Gal is usually expressed in the respiratory bronchiole and alveolus [53]. The low rate of laboratory confirmation for respiratory viral infections was the main limitation of this study, so prospective large-scale studies are required. In addition to respiratory viral test, serological test for influenza need to be taken considering low rate of viral isolation. Secondly, the classification of serotype is not widely used. Although we classified the groups based on comprehensive review of international and local data, some controversies might exist. Third, the yields of blood culture were quite low (2.1%). Given that this study was performed in the referral hospital, prior antibiotic use might have reduced the sensitivity of blood cultures, resulting in an underestimation of bacteremic pneumonia. Consequently, although a recent study estimated that about 4.5%–6.0% of invasive pneumococcal pneumonia can be attributed to influenza [54], we could not ascertain whether invasive pneumococcal pneumonia is more likely to concur following respiratory viral infections or not. To better detect bacteremic pneumonia, molecular diagnostic methods need to be considered. Finally, some uncontrolled confounding factors might exist, including vaccinations (influenza and pneumococcus), climate and socioeconomic levels.

In conclusion, serotypes 3 and 19A were the most prevalent among patients with pneumococcal pneumonia. Current pneumococcal vaccines (PPV23 and PCV13) should be effective against these serotypes. However, ICI serotypes were more likely to cause pneumococcal pneumonia after preceding respiratory viral illness, particularly in patients with either immunodeficiency or chronic lung diseases. ICI serotypes seemed to be transmitted more frequently secondary to increased colonization/carriage following epidemics of respiratory viruses. It is not clear why ICI serotypes would be more transmissible compared to others. Further clinical and experimental studies are warranted to clarify the pathogenesis.

## Supporting Information

File S1 **Tables S1 and S2.** Table S1. The coverage rates of pneumococcal vaccines, stratified by age group. Table S2. Serotype distribution of pneumococcal pneumonia: influenza epidemic periods versus non-epidemic periods.
(DOC)
