# Supplementary material for: Impact of Preceding Flu-Like Illness on the Serotype Distribution of Pneumococcal Pneumonia
Source: PLoS One. 2014 Apr 1;9(4):e93477. doi: 10.1371/journal.pone.0093477 (PMC3972234; doi:10.1371/journal.pone.0093477)
Supplement: File S1 — Tables S1 and S2. Table S1. The coverage rates of pneumococcal vaccines, stratified by age group. Table S2. Serotype distribution of pneumococcal pneumonia: influenza epidemic periods versus non-epidemic periods. (DOC) [file pone.0093477.s001.doc]

**Table S1.** The coverage rates of pneumococcal vaccines, stratified by age group.

| Serotype | No. of isolates (%) | | | | | |
| --- | --- | --- | --- | --- | --- | --- |
|  | ≤5 years | 6-18 years | 19-49 years | 50-64 years | ≥65 years | Total |
| PCV7 | 37 (36.3) | 8 (36.4) | 26 (18.1) | 55 (23.0) | 102 (24.8) | 228 (24.8) |
| PCV13 | 77 (75.5) | 14 (63.6) | 85 (59.0) | 144 (60.3) | 238 (57.8) | 558 (60.7) |
| PPV23 | 66 (64.7) | 16 (72.7) | 91 (63.2) | 141 (59.0) | 268 (65.0) | 582 (63.3) |
| 3 | 4 (3.9) | 1 (4.5) | 24 (16.7) | 26 (10.9) | 60 (14.6) | 115 (12.5) |
| 4 | 0 (0) | 0 (0) | 1 (0.7) | 3 (1.3) | 3 (0.7) | 7 (0.8) |
| 5 | 0 (0) | 0 (0) | 1 (0.7) | 0 (0) | 1 (0.2) | 2 (0.2) |
| 6A | 14 (13.7) | 1 (4.5) | 11 (7.6) | 28 (11.7) | 25 (6.1) | 79 (8.6) |
| 6B | 7 (6.9) | 4 (18.2) | 3 (2.1) | 10 (4.2) | 27 (6.6) | 51 (5.5) |
| 6C | 0 (0) | 0 (0) | 3 (2.1) | 1 (0.4) | 1 (0.2) | 5 (0.5) |
| 6D | 1 (1.0) | 0 (0) | 4 (2.8) | 9 (3.8) | 12 (2.9) | 26 (2.8) |
| 7B/7C/40 | 0 (0) | 0 (0) | 2 (1.4) | 0 (0) | 1 (0.2) | 3 (0.3) |
| 7F/7A | 0 (0) | 0 (0) | 0 (0) | 2 (0.8) | 1 (0.2) | 3 (0.3) |
| 8 | 0 (0) | 0 (0) | 1 (0.7) | 1 (0.4) | 0 (0) | 2 (0.2) |
| 9V/9A | 2 (2.0) | 0 (0) | 6 (4.2) | 4 (1.7) | 3 (0.7) | 15 (1.6) |
| 9N | 0 (0) | 0 (0) | 0 (0) | 1 (0.4) | 2 (0.5) | 3 (0.3) |
| 10A/39 | 0 (0) | 0 (0) | 1 (0.7) | 5 (2.1) | 2 (0.5) | 8 (0.9) |
| 10B | 0 (0) | 0 (0) | 0 (0) | 2 (0.8) | 1 (0.2) | 3 (0.3) |
| 11A/11E | 3 (2.9) | 2 (9.1) | 12 (12.0) | 12 (5.0) | 38 (9.2) | 67 (7.3) |
| 12F | 0 (0) | 0 (0) | 0 (0) | 0 (0) | 2 (0.5) | 2 (0.2) |
| 13 | 0 (0) | 0 (0) | 3 (2.1) | 3 (1.3) | 14 (3.4) | 20 (2.2) |
| 14 | 3 (2.9) | 0 (0) | 1 (0.7) | 7 (2.9) | 15 (3.6) | 26 (2.8) |
| 15F/15A | 5 (4.9) | 0 (0) | 4 (2.8) | 2 (0.8) | 6 (1.5) | 17 (1.8) |
| 15B | 0 (0) | 1 (4.5) | 3 (2.1) | 5 (2.1) | 7 (1.7) | 16 (1.7) |
| 15C | 0 (0) | 0 (0) | 0 (0) | 0 (0) | 1 (0.2) | 1 (0.1) |
| 16F | 0 (0) | 0 (0) | 1 (0.7) | 3 (1.3) | 0 (0) | 4 (0.4) |
| 17F/17A | 0 (0) | 0 (0) | 0 (0) | 1 (0.4) | 4 (1.0) | 5 (0.5) |
| 18C | 0 (0) | 0 (0) | 0 (0) | 0 (0) | 2 (0.5) | 2 (0.2) |
| 19F | 18 (17.6) | 1 (4.5) | 13 (9.0) | 18 (7.5) | 37 (9.0) | 87 (9.5) |
| 19A | 22 (21.6) | 4 (18.2) | 23 (16.0) | 33 (13.8) | 49 (11.9) | 131 (14.3) |
| 20 | 1 (1.0) | 0 (0) | 2 (1.4) | 4 (1.7) | 7 (1.7) | 14 (1.5) |
| 22F/22A | 1 (1.0) | 0 (0) | 2 (1.4) | 4 (1.7) | 9 (2.2) | 16 (1.7) |
| 23F | 7 (6.9) | 3 (13.6) | 2 (1.4) | 13 (5.4) | 15 (3.6) | 40 (4.4) |
| 23A | 0 (0) | 1 (4.5) | 0 (0) | 1 (0.4) | 6 (1.5) | 8 (0.9) |
| 24F/24A/24B | 1 (1.0) | 1 (4.5) | 1 (0.7) | 1 (0.4) | 1 (0.2) | 5 (0.5) |
| 25F/25A/38 | 0 (0) | 0 (0) | 0 (0) | 1 (0.4) | 0 (0) | 1 (0.1) |
| 28F/28A | 0 (0) | 0 (0) | 0 (0) | 0 (0) | 1 (0.2) | 1 (0.1) |
| 31 | 0 (0) | 0 (0) | 1 (0.7) | 3 (1.3) | 2 (0.5) | 6 (0.7) |
| 33F/33A/37 | 0 (0) | 1 (4.5) | 1 (0.7) | 2 (0.8) | 1 (0.2) | 5 (0.5) |
| 34 | 2 (2.0) | 0 (0) | 8 (5.6) | 7 (2.9) | 16 (3.9) | 33 (3.6) |
| 35F/47F | 0 (0) | 0 (0) | 0 (0) | 0 (0) | 1 (0.2) | 1 (0.1) |
| 35A/35C/42 | 0 (0) | 0 (0) | 0 (0) | 0 (0) | 1 (0.2) | 1 (0.1) |
| 35B | 1 (1.0) | 1 (4.5) | 4 (2.8) | 15 (6.3) | 18 (4.4) | 39 (4.2) |
| 36 | 0 (0) | 0 (0) | 0 (0) | 0 (0) | 2 (0.5) | 2 (0.2) |
| 41F/41A | 0 (0) | 0 (0) | 1 (0.7) | 0 (0) | 0 (0) | 1 (0.1) |
| 45 | 0 (0) | 0 (0) | 0 (0) | 1 (0.4) | 0 (0) | 1 (0.1) |
| Nontypeable | 10 (9.8) | 1 (4.5) | 5 (3.5) | 11 (4.6) | 18 (4.4) | 45 (4.9) |
| Group I | 8 | 1 | 4 | 10 | 17 | 40 |
| Group II | 2 (NCC1) | 0 | 1 (NCC3) | 1 (NCC3) | 1 (NCC2) | 5 |
| Total | 102 | 22 | 144 | 239 | 412 | 919 |

NCC, null capsule clade; ICI, infrequently colonizing but invasive serotypes; FCI, frequently colonizing and invasive serotypes; FCWI, frequently colonizing but weakly invasive serotypes

**Table S2. Serotype distribution of pneumococcal pneumonia: influenza epidemic periods versus non-epidemic periods.**

| Serotype, No. (%) | | Epidemic periods | Non-epidemic periods | *P* value |
| --- | --- | --- | --- | --- |
| ICI serotypes | | 25 (75.8) | 8 (24.2) | 0.01 |
| FCI serotypes | | 135 (49.6) | 137 (50.4) | 0.13 |
| FCWI serotypes | | 213 (53.0) | 189 (47.0) | 0.72 |
| Unclassified serotypes | | 120 (56.6) | 92 (43.4) | 0.35 |
| ICI serotypes | 4 | 4 (57.1) | 3 (42.9) | 0.85 |
|  | 5 | 2 (100) | 0 (0) | 0.50 |
|  | 7F/7A | 2 (66.7) | 1 (33.3) | 0.65 |
|  | 8 | 2 (100) | 0 (0) | 0.50 |
|  | 9V/9A | 12 (80) | 3 (20) | 0.06 |
|  | 12F | 1 (50) | 1 (50) | 0.92 |
|  | 18C | 2 (100) | 0 (0) | 0.50 |
| FCI serotypes | 3 | 64 (55.7) | 51 (44.3) | 0.64 |
|  | 14 | 10 (38.5) | 16 (61.5) | 0.16 |
|  | 19A | 61 (46.6) | 70 (53.4) | 0.08 |
| FCWI serotypes | 6A | 48 (60.8) | 31 (39.2) | 0.20 |
|  | 6B | 20 (39.2) | 31 (60.8) | 0.04 |
|  | 11A/11E | 34 (50.7) | 33 (49.3) | 0.70 |
|  | 15F/15A | 9 (52.9) | 8 (47.1) | 0.95 |
|  | 15B | 8 (50) | 8 (50) | 0.81 |
|  | 15C | 0 (0) | 1 (100) | 0.46 |
|  | 16F | 2 (50) | 2 (50) | 0.88 |
|  | 19F | 42 (48.3) | 45 (51.7) | 0.31 |
|  | 23F | 22 (55) | 18 (45) | 0.87 |
|  | 35B | 27 (69.2) | 12 (30.8) | 0.05 |
|  | 6D | 12 (46.2) | 14 (53.8) | 0.55 |
|  | 7B/7C/40 | 0 (0) | 3 (100) | 0.10 |
|  | 9N | 2 (66.7) | 1 (33.3) | 0.65 |
|  | 10A/39 | 3 (37.5) | 5 (62.5) | 0.48 |
|  | 10B | 2 (66.7) | 1 (33.3) | 0.65 |
|  | 13 | 12 (60) | 8 (40) | 0.65 |
|  | 17F/17A | 3 (60) | 2 (40) | 0.78 |
|  | 20 | 8 (57.1) | 6 (42.9) | 0.79 |
|  | 22F/22A | 12 (75) | 4 (25) | 0.13 |
|  | 23A | 5 (62.5) | 3 (37.5) | 0.73 |
|  | 24F/24A/24B | 4 (80) | 1 (20) | 0.38 |
|  | 25F/25A/38 | 1 (100) | 0 (0) | 0.35 |
|  | 28F/28A | 1 (100) | 0 (0) | 0.35 |
|  | 31 | 2 (33.3) | 4 (66.7) | 0.42 |
|  | 33F/33A/37 | 5 (100) | 0 (0) | 0.07 |
|  | 34 | 18 (54.5) | 15 (45.5) | 0.92 |
|  | 35F/47F | 1 (100) | 0 (0) | 0.35 |
|  | 35A/35C/42 | 1 (100) | 0 (0) | 0.35 |
|  | 36 | 1 (50) | 1 (50) | 0.92 |
|  | 41F/41A | 1 (100) | 0 (0) | 0.35 |
|  | 45 | 1 (100) | 0 (0) | 0.35 |
|  | Nontypeable | 23 (51.1) | 22 (48.9) | 0.76 |
|  | Group I | 20 | 20 | - |
|  | Group II | 3^*^ | 2^†^ | - |
| Total | | 493 (53.6) | 426 (46.4) |  |

^*^ One each isolate of null capsule clade (NCC) 1, NCC2, and NCC3

^†^ One NCC1 isolate and one NCC3 isolate

ICI, infrequently colonizing but invasive serotypes; FCI, frequently colonizing and invasive serotypes; FCWI, frequently colonizing but weakly invasive serotypes
